# Plasmid-mediated quinolone resistance determinants in clinical bacterial pathogens isolated from the Western Region of Ghana: a cross-sectional study

**DOI:** 10.11604/pamj.2022.43.207.33734

**Published:** 2022-12-27

**Authors:** Andrews Kwabena Sah, Patrick Kwame Feglo

**Affiliations:** 1 Department of Clinical Microbiology, School of Medicine and Dentistry, Kwame Nkrumah University of Science and Technology, Kumasi, Ghana,; 2 Laboratory Unit, Prestea Government Hospital, Prestea, Ghana

**Keywords:** Antimicrobial resistance, quinolones, plasmid-mediated quinolone resistance genes, Ghana

## Abstract

**Introduction:**

quinolones are critically important antibiotics that are reserved for treating very severe infections caused by multidrug-resistant bacterial pathogens. However, their indiscriminate uses have resulted in an increased number of resistant strains in many parts of the world including Ghana. We determined the quinolone resistance profile of Gram-negative bacterial pathogens and characterized the underlying molecular determinants of resistance.

**Methods:**

Gram-negative pathogens obtained from clinical specimens at three hospital laboratories were tested for resistance to quinolones and other commonly used antibiotics. ESBL production among the Enterobacterial isolates was confirmed using the combined disc diffusion method. We then used PCR to determine seven types of plasmid-mediated quinolone resistance genes present in the isolates resistant to nalidixic acid and ciprofloxacin.

**Results:**

in this study, 29.5% of the isolates were resistant to ciprofloxacin, with the highest of 50% among E. coli resistance to the other quinolones was levofloxacin (24.4%), norfloxacin (24.9%), and nalidixic acid (38.9%). Significant proportions of the quinolone-resistant isolates were ESBL producers (P-values < 0.001). The aac(6´)-Ib-cr, qnrS, oqxA, and qepA genes were present in 43 (89.6%), 27 (56.3%), 23 (47.9%), and one (2.1%) of the isolates, respectively. None of the isolates tested positive to qnrA, qnrB, and oqxB genes. The presence of the aac(6´)-Ib-cr gene positively correlated with resistance to ceftriaxone, cefotaxime, and gentamicin (P-values < 0.05).

**Conclusion:**

high proportions of Gram-negative bacterial isolates were resistant to quinolones and most of these isolates possessed multiple PMQR genes. There is a need to implement measures to limit the spread of these organisms.

## Introduction

Antibiotics are essential drugs for controlling and preventing morbidity and mortality from infection caused by bacteria. However, rising resistance levels in nearly all bacteria types that cause illnesses have substantially reduced their effectiveness and threatened a return to the pre-antibiotic era [1]. This has led to increased length of hospital stays, cost of healthcare, morbidity and death [2]. Quinolones are an important group of these essential drugs and were introduced into clinical practices in the mid-1980s. They are well known for their ease of absorption following oral administration, with higher concentrations in the urinary tract. This often makes them the drug of choice for urinary tract infections. Newer generations such as ciprofloxacin are mostly preferred in treating infections such as enteric fevers to other groups which are required to be administered parenterally, including the cephalosporins and aminoglycosides [3]. Quinolones are also one of the major options for the treatment of infections caused by dangerous and multidrug-resistant bacteria [1]. In Ghana, quinolones were introduced for the treatment of bacterial infections due to increased resistance levels in bacterial pathogens to commonly used, less expensive, and readily available antibiotics such as the penicillins, sulfonamides and first and second-generation cephalosporins [1,4]. However, the recurrence of bacterial resistance to this family of antibiotics is limiting their use globally, whiles alternatives are limited. Urgent measures are therefore required to reduce the spread of resistant bacteria. This will help reduce the already existing pressure on health systems in resource-limited countries and promote the achievement of the Sustainable Development Goals (SDGs) [2].

Resistance to the quinolones is generally higher in developing countries. A systematic review in 2015 recorded levels as low as 2% in USA and Ireland, compared to 80% in Nigeria [5]. In Ghana, up to 50% resistance to ciprofloxacin was reported among pathogens obtained from wound and blood cultures [6,7]. In particular, there has been increasing reports of reduced susceptibility of *Salmonella* species to fluoroquinolones [4,8,9]. Resistance to quinolone antibiotics is mediated by chromosomal mutations and the acquisition of transferable plasmid genes [3,10,11]. Several forms of plasmid-mediated quinolone resistance (PMQR) genes have been described and most of these have been detected in clinical isolates [3]. These genes facilitate the selection and spread of quinolone-resistant bacterial strains rapidly and pose a greater threat to public health [10,12]. Studies in most countries have determined the prevalence and characteristics of the various resistant determinants in clinical isolates. In Ghana, there is a dearth of studies on quinolone resistance determinants in clinical isolates and the few studies are centered and localized to teaching hospital in the two big cities in the country; Accra and Kumasi. This study aimed to determine the molecular determinants that mediate quinolone resistance among Gram-negative bacterial pathogens in the Western Region of Ghana. Data obtained from the study are essential to inform policy on antibiotic stewardship in the region and to monitor the effectiveness of existing interventions.

## Methods

**Study design, sites, and populations**: a prospective cross-sectional study was conducted in the Western Region of Ghana from October 2020 to February 2021. The region is situated in the Southwestern part of Ghana. It shares borders with the Central Region in the East, Western North Region in the North, La Cote d´Ivoire in the West, and the Gulf of Guinea (Atlantic Ocean) in the South [13]. The region is divided into fourteen administrative divisions, including one Metropolitan, seven Municipalities, and six Districts [14]. Clinical specimens of patients conducting microbiology tests at the Sekondi Public Health Laboratory (SPHL), situated in the Western Regional capital; Tarkwa Municipal Hospital (TMH), in a semi-urban city; and Prestea Government Hospital (PGH), in a rural town were obtained for the study. Gram-negative bacterial pathogens isolated from Mid-stream urine, blood and wound swab specimens were included in the study.

**Bacterial isolation and identification**: the specimen were cultured using conventional techniques [15]. We used cysteine-lactose-electrolyte-deficient (CLED) agar for culturing the urine specimens. Blood and wound specimens were cultured on MacConkey agar and blood agar. The inoculated plates were incubated aerobically at 35-37^°^C and examined after 18-24 hours for bacterial growth. For the urine cultures, a count of 10^5^ CFU/mL or more bacterial growth was considered significant for further investigations. The bacterial pathogens were identified using conventional methods including their Gram staining reactions, colonial characteristics, and biochemical properties.

**Antibiotic susceptibility testing**: Gram-negative isolates were selected for antimicrobial susceptibility testing using the modified Kirby-Bauer disc diffusion method in accordance with the Clinical and Laboratory Standards Institute (CLSI) guidelines [16]. The antimicrobials tested included ciprofloxacin (5µg), levofloxacin (5µg), amikacin (30µg), piperacillin-tazobactam (110µg), gentamicin (10µg) ceftazidime (30µg), meropenem (10µg). nalidixic acid (30µg), norfloxacin (10µg), ampicillin-sulbactam (20µg), amoxicillin-clavulanic acid (30 µg), cefotaxime (30µg), nitrofurantoin (300µg), ceftriaxone (30µg), and trimethoprim-sulfamethoxazole (25µg). Among the *Enterobacteriaceae*, isolates that tested resistant to cefotaxime, ceftazidime, or ceftriaxone were suspected to be ESBL producers [16], and were confirmed using the combination disc method [16].

**Detection of plasmid-mediated quinolone resistance genes using polymerase chain reaction (PCR)**: following the manufacturer´s instructions, we extracted plasmid DNA from 48 isolates resistant to ciprofloxacin and nalidixic acid using ZymoPURE^™^ Plasmid Miniprep kit (Zymo Research Corporation). We used qualitative conventional polymerase chain reaction (PCR) carried out on TC-512 (Techne, UK) thermocycler to amplify seven plasmid-mediated quinolone resistance genes. The genes and the primers used were *qnrA* (forward 5´- AGAGGATTTCTCACGCCAGG-3´ and reverse 5´- GCAGCACTATKACTCCCAAGG-3´), *qnrB* (forward 5´ GGMATHGAAATTCGCCACTG-3´ and reverse 5´- TTTGCYGYYCGCCAGTCGAA-3´), *qnrS* (forward 5´- GCAAGTTCATTGAACAGGCT-3´ and reverse 5´- TCTAAACCGTCGAGTTCGGCG-3´), *qepA* (forward 5´- CTGCAGGTACTGCGTCATG-3´ and reverse 5´- CGTGTTGCTGGAGTTCTTC-3´), *OxqA* (forward 5´-GACAGCGTCGCACAGAATG-3´ and reverse 5´-GGAGACGAGGTTGGTATGGA-3´), *oxqB* (forward 5´-CGAAGAAAGACCTCCCTACCC-3´ and reverse 5´-CGCCGCCAATGAGATACA-3´), and *aac(6´)-Ib-cr* (forward 5´-TTGCGATGCTCTATGAGTGGCTA-3´ and reverse 5´-CTCGAATGCCTGGCGTGTTT-3´) [17]. A 25 µl reaction volume consisting of 9µl nuclease-free water, 0.5 µl of 10 µm forward primer, 0.5 µl of 10 µm reverse primer, 12.5 µl of “One-Taq Quick Load 2X Mater Mix with Standard Buffer” and 2.5 µl of template DNA was set up. The PCR was run using the following cycling conditions: initial denaturation at 94^°^C for 30 seconds; 30 cycles of denaturation at 94^°^C for 30 seconds, annealing for 30 seconds at 55^°^C for *qnrA, qnrB, qnrS and aac(6´)-1b-cr*and at 54^°^C for *qepA, oqxA* and *oqxB*, and extension at 68^°^C for 60 seconds; final extension at 68^°^C for 5 minutes; and holding at 4^°^C till removed from the thermocycler. The amplicons were resolved using electrophoresis on 1.5% agarose gel at 5V/cm for 60 minutes.

**Data analysis**: the data generated from the study were captured into Microsoft Excel 2013 spreadsheet and exported into IBM SPSS Statistics 23 for analysis. The antibiotic sensitivity results (zone diameters) were captured, analyzed and interpreted using the 2020 version of WHONET software. The results were summarized into tables and charts. Categorical variables were presented as frequencies and proportions (%), and continuous variables as mean and median. A chi-square test was used to compare the categorical variables while a student T-Test was used for continuous variables. A p-value of ≤0.05 was considered significant.

**Ethical considerations**: the Committee on Human Research Publication and Ethics of the School of Medicine and Dentistry at Kwame Nkrumah University of Science and Technology approved the study protocol with reference number CHRPE/AP/302/20. The authorities of all the participating facilities also granted permission for sample collection and access to laboratory units. Informed consent was obtained from the participants by the health professional collecting the samples after adequate information about the project was explained to them in accordance with the Declaration of Helsinki.

## Results

**Demographic data**: between October 2020 and February 2021, 820 specimens were collected from 807 patients (591 females and 216 males). The specimens included urine 538 (65.6%), blood 180 (22%), and wound 102 (12.4%). Most of the specimens 376 (44.8%) were collected at Sekondi Public Health Laboratory, 203 (24.8%) at Tarkwa Municipal Hospital, and 237 (28.9%) at Prestea Government Hospital (Table 1). The ages of the participants ranged from 2 days to 94 years, with a median age of 28. Most of the participants, 572 (70.1%), sought outpatient services, whereas in-patients constituted 235 (19.1%).

**Table 1:** the distribution of bacterial pathogens isolated from the Western Region of Ghana, stratified by gender, patient category, age, specimen type, and testing laboratory

Variable	Isolates, n (%)								
	*Escherichia coli*70 (25.7)	*Klebsiella spp*.71(26.1)	*Enterobacter spp*.28 (10.3)	*Citrobacter spp*.19(7.0)	*Salmonella spp*.12 (4.4)	*Proteus spp*.12(4.4)	*Pseudomonas aeruginosa*38 (14.0)	*Others 22 (8.1)	Total 272 (100)
**Gender**									
Male	11(15.0)	20(27.4)	5(6.8)	6(8.2)	8(11.0)	2(2.7)	11(15.1)	10(13.6)	73(100)
Female	59(29.6)	51(25.6)	23(11.6)	13(6.5)	4(2.0)	10(5.0)	27(13.6)	12(6.3)	199(100)
**Patient**									
Out-Patient	51(26.8)	50(26.3)	25(13.2)	12(6.3)	6(3.2)	9(4.7)	26 (13.7)	11(5.8)	190(100)
In-Patient	19(23.2)	21(25.6)	3(3.7)	7(8.5)	6(7.3)	3(3.7)	12(14.6)	11(13.4)	82(100)
**Age**									
Child	2(6.1)	8(24.2)	1(3.0)	5(15.1)	10(30.3)	4(12.1)	1(3.0)	2(6.1)	33 (100)
Adult	68 (28.5)	63 (26.4)	27 (11.3)	14 (5.9)	2 (0.8)	8 (3.3)	37 (15.5)	20 (8.4)	239 (100)
**Specimen**									
Urine	56 (34.8)	48 (29.8)	23 (14.3)	10 (6.2)	0	3 (1.9)	16 (9.9)	5 (3.1)	161 (100)
Blood	3 (10.3)	6 (20.7)	0	4(13.8)	12(41.8)	0	1(3.4)	3(10.3)	29(100)
Wound	11(13.4))	17(20.7)	5(6.1)	5(6.1)	0	9(10.9)	21(25.6)	14 (17.1)	82(100)
**Laboratory**									
SPHL	35(28.9)	35(28.9)	8(6.6)	9(7.4)	12(9.9)	6(5.0)	5(4.1)	11(9.1)	121(100)
TMH	19(31.1)	16(26.2)	4(6.6)	1(1.6)	0	2(3.3)	11(18.0)	8(13.1)	61 (100)
PGH	16(17.8)	20(22.2)	16(17.8)	9(10)	0	4(4.4)	22(24.4)	3(3.3)	90(100)

n=number of isolates, SPHL= Sekondi Public Health laboratory; TMH= Tarkwa Municipal Hospital; PGH= Prestea Government Hospital * Include six strains of Acinetobacter spp., eight of Aeromonas spp., three of Providencia spp., two of Hafnia spp., two of Morganella spp., and one Serratia sp.

**Bacteria isolated:** among the 807 participants, the samples from 258 were culture-positive for Gram-negative bacteria, giving a prevalence of 32.0%. These 258 specimens yielded 272 bacterial isolates in total, out of which 254 were used for further analysis (Table 1). Majority of the isolates were *Klebsiella spp*. 71 (26.1%) and *E. coli* 70 (25.7%).

**Quinolone antimicrobial resistance pattern of the isolates**: antimicrobial susceptibility testing was performed on 254 isolates. The proportions of the isolates that were resistant to the four tested quinolones in descending order were nalidixic acid 86/221 (38.9%), ciprofloxacin 75/254 (29.5%), norfloxacin 55/221 (24.9%), and levofloxacin 62/254 (24.4%). Among the isolates, the highest proportions of resistance to the quinolones were recorded in *E. coli* followed by *Klebsiella spp*., while *Salmonella spp*. and *Proteus spp*. had the least resistance proportions (Table 2). The proportions of *E. coli* and *Klebsiella spp*. resistant to the tested quinolones were compared; the results indicated that *E. coli* isolates had higher resistance proportions than *Klebsiella spp*. and the differences were statistically significant (p-values < 0.05). Among patient groups and sexes there were no significant differences in resistance proportions to any of the quinolones tested (P-values > 0.05). In this study, geographical differences in quinolone resistance were observed. Although, isolates from Sekondi Public Health Laboratory and Tarkwa Municipal Hospital showed no significant differences in resistance to the tested quinolones, they had significantly higher levels of resistance to all the four quinolones than isolates from Prestea Government Hospital (P values < 0.05) (data not shown).

**Table 2:** antibiotic resistance pattern of Gram-negative pathogens isolated from patients at three laboratories in the Western Region of Ghana

Variable	Isolates								
	*Escherichia coli* n=70	*Klebsiella spp*. n=69	*Enterobacter spp*. n=27	*Citrobacter spp*. n=18	Salmonella spp. n=12	*Proteus spp*. n=12	*Pseudomonas aeruginosa* n=29	Others*β n=17	Total n=254
**Antibiotic resistance, n (%)**									
**Ciprofloxacin**	35(50)	20(29)	3(11.1)	4(22.2)	0	0	7(24.5)	6(35.3)	75(29.5)
**Nalidixic acid**	47(67.1)	21(30.4)	7(18.5)	5(38.9)	1(8.4)	2(16.7)	-	3(27.3)	86(38.9)
**Levofloxacin**	32(45.7)	14(20.3)	2(7.4)	2(11.1)	0	0	6(20.7)	6(35.3)	62(24.4)
**Norfloxacin**	*35(50)*	15(21.7)	2(7.4)	2(11.1)	0	0	-	2 (18.2)	55 (24.9)
**Amikacin**	1 (1.4)	1 (1.4)	0	0	0	0	1 (3.4)	2 (11.8)	5 (2.0)
**Gentamicin**	21 (30.0)	23 (33.3)	7 (25.9)	4 (22.2)	1 (8.4)	0	6 (20.7)	6 (35.3)	68 (26.9)
**Piperacillin/ tazobactam**	19 (27.1)	18 (27.5)	4 (14.8)	2 (11.1)	0	0	8 (27.6)	5 (29.4)	57 (22.4)
**Amoxicillin/ clavulanic acid**	38 (54.3)	35 (50.7)	17 (63.0)	7 (38.7)	0	1 (8.3)	-	7 (53.8)	105 (47.1)
**Ampicillin/sulbactam**	30 (42.9)	30 (43.5)	15 (55.6)	5 (27.8)	0	0	-	4 (36.4)	84 (38.1)
**Ceftriaxone**	34 (48.6)	33 (47.8)	10 (37.0)	9 (50.0)	2 (16.7)	0	-	5 (45.5)	94 (42.5)
**Cefotaxime**	34 (48.6)	33 (47.8)	10 (37.0)	9 (50.0)	2 (16.7)	0	-	5 (45.5)	94 (42.5)
**Ceftazidime**	14 (20.0)	19 (27.5)	7 (25.9)	3 (16.7)	1 (8.4)	0	8 (27.6)	5 (29.4)	57 (22.4)
**Trimethoprim/ sulfomethoxazole**	53 (75.7)	50 (72.5)	14 (51.9)	14 (77.8)	0	6 (50.0)	-	5 (45.5)	143 (64.7)
**Meropenem**	0	3 (4.3)	1 (3.7)	0	0	0	5 (17.2)	3 (17.6)	12 (4.7)
**Nitrofurantoin	3 (4.3)	20 (41.7)	10 (51.9)	4 (36.4)	-	1 (25.0)	-	2 (50.0)	47 (30.1)
***ESBL	34(48.6)	27(39.1)	7(25.9)	6(33.3)	2(16.7)	0	-	1(16.7)	77(46.5)

βInclude 6 strains of Acinetobacter spp., 4 Aeromonas spp., 3 Providencia spp., 2 Morganella spp., 1 Serratia sp. and 1 Hafnia sp. * Acinetobacter spp. was tested with ciprofloxacin, levofloxacin, Amikacin, gentamicin, piperacillin/tazobactam, ceftazidime, and meropenem only. **Tested against only urine isolates, *** Enterobacteriaceae isolates (except the Serratia sp) were tested

**Resistance patterns of the isolates to other antimicrobials**: the isolates were tested against eleven other antimicrobials. The highest proportion of the isolates were resistant to trimethoprim sulfamethoxazole 143 (64.7%), followed by amoxicillin clavulanic acid 103 (46.6%), ceftriaxone 95 (42.5%), and cefotaxime 94 (42.5%), the least proportions of resistance were to amikacin 5 (2.0%) and meropenem 12 (4.7%) (Table 2).

**Extended spectrum beta-lactamase (ESBL) production as a predictor of resistance to quinolones**: we tested 215 *Enterobacteriaceae* isolates for ESBL production and 77 (35.8%) were confirmed as ESBL producers. The highest proportion of ESBL producers was observed among *E. coli* 34/70 (48.6%), followed by *Klebsiella spp*. 27/69 (39.1%), and *Citrobacter spp*. 6/19 (31.6%). None of the *Proteus spp*. was ESBL producer. There was a link between quinolone-resistance and ESBL production. The proportions of ESBL producing isolates and the non-ESBL producing isolates resistant to the tested quinolones were compared. Isolates that produce ESBL were more likely to be resistant to the quinolone antibiotics than their non-ESBL producing counterparts (odd ratios > 43, P-values < 0.0001).

**Figure 1:**
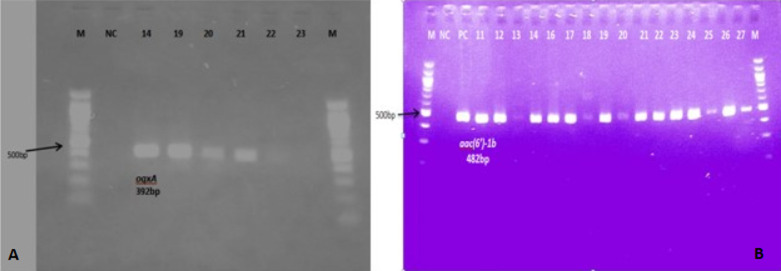
agarose gel electrophoresis of PCR products; A) oqxA; B) aac(6´)-Ib-cr

**Plasmid-mediated quinolone resistant genes**: we screened forty-eight (48) samples, made up of 27 strains of *E. coli*, 16 strains of *Klebsiella spp*., four of *Citrobacter spp*., and one *Enterobacter spp*. for PMQR genes using conventional PCR. Out of this, 46 (95.8%) tested positive for at least one of the PMQR genes, 30 (62.5%) samples contained multiple genes with 15 (31.3%) harboring three (3) genes. The plasmid genes detected were *aac(6´)-Ib-cr*43 (89.6%), *qnrS*27 (56.3%), *oqxA*23 (47.9%), and *qepA* one (2.1%). We did not detect the *qnrA, qnrB*, and *oqxB* genes in any of the samples tested. Figure 1 shows examples of gel images of PCR products captured. Among the 27 strains of *E. coli*. tested, *aac(6´)-Ib-cr, qnrS, oqxA* and *qepA* were present in 22 (81.5%), 19 (70.4%), 17 (63.0%), and one (3.7%) respectively. All the *Klebsiella spp., Citrobacter spp*., and *Enterobacter sp*. tested possessed *aac(6´)-Ib-cr gene*. One (25%) of *Citrobacter*species possessed *qnrS* and *oqxA* genes. Among the *Klebsiella* strains, six (37.5%) and five (3.3%) possessed *qnrS* and *oqxA* genes respectively while among *Citrobacter spp. qnrS*, one (25%) and *oqxA*, one (25%) genes were present. The *Enterobacter spp*. also possessed *qnrS* gene.

The presence of PMQR genes was investigated to determine whether it affects susceptibility to antibiotics other than quinolones (Table 3). We observed that the presence of *qnrS and oqxA* genes did not correlate with the proportion of the isolates that were resistant to any of the antimicrobials tested (p-values > 0.05). However, the presence of *aac(6´)-Ib-cr* gene positively correlated to the proportions of the isolates that were resistant to cefotaxime (P-value=0.006), ceftriaxone (p-value=0.018), and gentamicin (p-value=0.020).

**Table 3:** plasmid-mediated quinolone-resistant genes as predictors of resistance to non-quinolone antibiotics

Variables	PMQR genes								
Antibiotics	*qnrS*			*oqxA*			*aac(6´)-Ib-cr*		
	Present n=27	Absent n =21	P-value	Present n=23	Absent n=25	P-value	Present n=43	Absent n=5	P-value
**CTR**	23(85.2)	17(81.0)	0.927	19(82.6)	21(84.0)	0.992	38(88.4)	2(40.0)	**0.018**
**CTX**	23(85.2)	17(81.0)	0.715	19(82.6)	21(84.0)	1.000	38(88.4)	2(40.0)	**0.006**
**CAZ**	8(29.6)	11(52.8)	0.277	8(34.8)	11(44.0)	0.676	19 (57.6)	0	0.056
**AMK**	0	1(4.8%)	0.438	0	1(4.0)	1.000	1(2.3)	0	1.000
**GEN**	12(44.4)	13(61.9)	0.259	10(43.5)	15(60.0)	0.386	25 (58.1)	0	**0.020**
**AMC**	22(81.5)	17 (81.0)	0.493	19 (82.6)	20 (80.0)	0.990	36 (83.7)	3 (60.0)	0.325
**TZP**	11(47.7)	11 (52.4)	0.587	12 (52.2)	10 (40.0)	0.630	21 (55.3)	1 (25.0)	0.249
**SAM**	18(66.7)	16(76.2)	0.499	16 (69.6)	18 (72.0)	0.548	31(72.1)	3(60.0)	0.111
**MEM**	0	2(9.5)	0.128	0	2(8.0)	0.229	2(4.7)	0	0.830
**SXT**	25(92.6)	16(76.2)	0.215	21(91.3)	20(80.0)	0.419	38 (88.4)	3 (60.0)	0.089
**NIT**	2(7.4)	4(19.0)	0.479	2(8.7)	4(16.0)	0.910	6(18.2)	0	0.418

PMQR-Plasmid-Mediated Quinolone Resistance, AMK-Amikacin; GEN-Gentamicin; TZP-Piperacillin tazobactam; SAM-Ampicillin sulbactam; AMC-Amoxicillin clavulanate; CTR-Ceftriaxone; CTX-Cefotaxime; CAZ-Ceftazidime; SXT-Trimethoprim sulfamethoxazole; NIT-Nitrofurantoin; MEM-Meropenem.

## Discussion

Antimicrobial resistance has emerged as an important threat to human and animal health worldwide, calling for intensive research to elucidate the complexity of underlying causes and possible ways of reducing, stopping, and possibly reversing the rate of its development [18,19]. In this study, we studied the antibiotic resistance patterns of 254 clinical Gram-negative bacterial isolates collected from three hospitals in the Western Region of Ghana. The presence of plasmid-mediated quinolone resistance genes was determined in selected ciprofloxacin and nalidixic acid-resistant isolates. The overall prevalence of Gram-negative bacterial pathogens from all the clinical specimens was 32.0% in the current study. This is similar to the finding of a study from Ethiopia where 34.7% of specimens had Gram-negative bacteria recovered from them [20]. However, it is higher when compared to results of studies from Mexico and Nepal, where prevalence of 19.1% and 17%, respectively, were reported [21,22]. These observed differences could be attributed to the differences in study settings, choice of study populations, sources of samples, and sample sizes. Among the thirteen different Gram-negative bacterial species identified, *E. coli* and *Klebsiella* species were the most dominant. They constituted 51.8% of the total isolates, as shown in Table 1. This is similar to the results of several studies, showing that, among Gram-negative bacterial pathogens, *E. coli* and *Klebsiella spp*. are frequent cause of infections in humans. These pathogens are becoming difficult to treat due to the development of resistance to the readily available and less costly drugs used by health professionals to treat them [20,22-24].

In this study, resistance patterns of thirteen bacterial species to fifteen antibiotics belonging to six different antibiotic classes were studied. Generally, high proportions of the isolates were resistant to the commonly prescribed antibiotics except for amikacin and meropenem where resistance proportions were below 5%. This result is similar to the findings of Agyepong and colleagues who found resistance proportions of Gram-negative bacteria isolated from a teaching hospital in Ghana at 3.5% and 2.5% to amikacin and meropenem, respectively [23]. Meropenem and Amikacin are relatively expensive making them not readily available, limiting their use in the study population. In addition, these drugs are recently introduced into the Ghanaian market and are mostly used as a last resort for treating multidrug-resistant infections. This has possibly led to relatively low natural selection and hence low development of resistance among the isolates. However, the emergence of resistance to these drugs especially, the carbapenems is worrying [25] and if unchecked can become a greater public health challenge in the future. Quinolone antibiotics are widely used to treat various forms of bacterial infections since being introduced in Ghana in the past two decades [4]. Due to their lower prices, accessibility, and ease of administration, these drugs have extensively been abused [26]. In a recent multicenter point prevalence survey across seven hospitals in Ghana, ciprofloxacin was among the top five most prescribed antibiotics to in-patients. The survey found 10% of these prescriptions to have no documented reasons [27]. The increased and inappropriate use of these drugs [28] has resulted in an increased selection for resistant bacteria strains. In this study, overall proportion of isolates that were resistant to ciprofloxacin was found to be 29.5% ranging from 0% in *Proteus spp*. and *Salmonella spp*. to 50% in *E. coli*. This is similar to the results of other studies where resistance of Gram-negative bacterial pathogens to ciprofloxacin were found to be 25% [29] and 26.7% [30]. Although, the proportions of resistance to ciprofloxacin were low compared to other studies [20,23,31], it is relatively high compared to findings from other countries, especially those in Europe and North America [32,33]. These observed differences were probably due to differences in geographical distribution of resistant bacterial strains [34], study population, study design [35], existing drug prescription policies, AMR surveillance, and antimicrobial stewardship programs [26,36]. Infection with a quinolone-resistant and/or ESBL producing organism has significant public health implications. These infections have limited treatment options, making it difficult for clinicians to prescribe successful treatment. They also make empiric prescriptions perilous since they are often associated with poorer treatment outcomes, including increased treatment failure, mortality, and morbidity [26,37]. They also increase the pressure on health facilities, which are often inadequate in our settings, due to increased hospital stays and the need for additional procedures due to complications [38]. Invariably, these translate into a higher social and economic burden on individuals, families, and the community.

In the present study, four PMQR genes were detected in decreasing order: *aac(6´)-Ib-cr*43 (89.6%), *qnrS*27 (56.2%), *oqxA*23 (47.9%), and *qepA* one (2.1%) among ciprofloxacin and nalidixic acid resistant isolates. The predominance of the *aac(6´)-Ib-cr* gene is supported by the work of Attipoe *et al*. involving *E. coli* isolated from 18 testing laboratories across Ghana. In their work, all 29 isolates tested were positive for the *aac(6´)-Ib-cr* gene. They also found a larger proportion of *E. coli* isolates to possess *qnrS*26 (89.6%), *oqxA*19 (65.5%), *qnrA*16 (55.1%), and *qnrB*15 (55.1%) [39]. Contrarily, the present study did not detect *qnrA, qnrB* and *oqxB* in the isolates. This may be due to differences in the geographical distribution of PMQR genes. Other studies in Nigeria [40] and Iran [41] confirmed the predominance of the *aac(6´)-Ib-cr* gene. These reports indicate the widespread presence of this quinolone-resistance gene.

In this study, no correlation was observed between the presence of *qnrS* and *oqxA* genes and the level of resistance to the non-quinolone antibiotics tested. This implies that, the presence of these genes alone may not be enough to affect the efficacy of the non-quinolone antibiotics. Isolates possessing *aac(6´)-Ib-cr* genes were found to have significant levels of resistance to cefotaxime (P-value = 0.006), ceftriaxone (P-value = 0.018), and gentamicin (P-value = 0.020) as compared to their *aac(6´)-Ib-cr* negative counterparts. No correlation was found with the other antibiotics. The enzyme aminoglycoside acetyltransferase, encoded by the *aac(6´)-Ib-cr* gene is originally known to confer resistance to the aminoglycosides (tobromycin, kanamycin, amikacin and gentamicin). Hence resistance to gentamycin in the presence of *aac(6´)-Ib-cr* gene is expected. The *aac*(6´)-Ib-cr gene is found in various integrons, especially on IncF11 plasmids that express CTX-M-15 [12]. CTX-M-15 is a widespread ESBL that confers resistance to third generation cephalosporin such as ceftriaxone and cefotaxime. Hence, isolates with *aac(6´)-Ib-cr* genes are likely to be resistance to third generation cephalosporin. These may explain the observed association between the presence of *aac(6´)-Ib-cr* gene and resistance to ceftriaxone and cefotaxime [12].

**Limitations of this study**: sequencing of PMQR genes identified in the study was not done to determine the allelic forms present in the study areas. Minimum inhibition concentration (MIC) determination for quinolone antibiotics was not done. MIC could have helped to determine if there is difference in levels of resistance to quinolone among the isolates positive and negative for PMQR genes. In addition, this is a laboratory based study so risk factors such as previous admission to hospital, length of stay at the hospital, presence of indwelling devices, and history of antibiotic use could not be assessed.

## Conclusion

In this study, high proportion of quinolone-resistant Gram-negative bacterial pathogens possessed multiple horizontally transferable PMQR genes. These genes promote the selection of highly quinolone-resistant bacterial strains that when spread can pose serious threat to public health in the region and Ghana. It is therefore recommended to the regional health authorities and stakeholders to institute measures to ensure adequate antimicrobial stewardship and surveillance, adequate infection prevention and control, and good prescription behavior among health workers to prevent further spread of quinolone resistant bacterial pathogens.

### What is known about this topic

- The role of plasmid-mediated quinolone resistance genes in the development of bacterial resistance to quinolone antibiotics;
- The circulation of plasmid-mediated quinolone genes in the gram-negative bacterial population of Ghana;
- The production of extended-spectrum beta-lactamase as predictor of quinolone antimicrobial resistance in gram-negative bacterial.

### What this study adds

- The prevalence of aac(6´)-Ib-cr, qnrS, oqxA, and qepA among quinolone-resistant gram-negative bacterial pathogens isolated from the Western Region of Ghana;
- The prevalence of quinolone antimicrobial resistance among gram-negative bacterial pathogens isolated from the Western Region of Ghana.

## References

[1] Collignon PC, Conly JM, Andremont A, McEwen SA, Aidara-Kane A (2016). World Health Organization ranking of antimicrobials according to their importance in human medicine: a critical step for developing risk management strategies to control antimicrobial resistance from food animal production. Clin Infect Dis.

[2] Murray CJ, Ikuta KS, Sharara F, Swetschinski L, Robles Aguilar G, Gray A (2022). Global burden of bacterial antimicrobial resistance in 2019: a systematic analysis. The Lancet.

[3] Kim ES, Hooper DC (2014). Clinical importance and epidemiology of quinolone resistance. Infect Chemother.

[4] Eibach D, Al-Emran HM, Dekker DM, Krumkamp R, Adu-Sarkodie Y, Cruz Espinoza LM (2016). The emergence of reduced ciprofloxacin susceptibility in *Salmonella* enterica causing bloodstream infections in rural Ghana. Clin Infect Dis.

[5] Fasugba O, Gardner A, Mitchell BG, Mnatzaganian G (2015). Ciprofloxacin resistance in community-and hospital-acquired Escherichia coli urinary tract infections: a systematic review and meta-analysis of observational studies. BMC Infect Dis.

[6] Janssen H, Janssen I, Cooper P, Kainyah C, Pellio T, Quintel M (2018). Antimicrobial-resistant bacteria in infected wounds, Ghana, 20141. Emerg Infect Dis.

[7] Groß U, Amuzu SK, de Ciman R, Kassimova I, Groß L, Rabsch W (2011). Bacteremia and antimicrobial drug resistance over time, Ghana. Emerg Infect Dis.

[8] Acheampong G, Owusu M, Owusu-Ofori A, Osei I, Sarpong N, Sylverken A (2019). Chromosomal and plasmid-mediated fluoroquinolone resistance in human Salmonella enterica infection in Ghana. BMC Infect Dis.

[9] Andoh LA, Ahmed S, Olsen JE, Obiri-Danso K, Newman MJ, Opintan JA (2017). Prevalence and characterization of *Salmonella* among humans in Ghana. Trop Med Health.

[10] Martínez-Martínez L, Pascual A, Jacoby GA (1998). Quinolone resistance from a transferable plasmid. The Lancet.

[11] Correia S, Poeta P, Hébraud M, Capelo JL, Igrejas G (2017). Mechanisms of quinolone action and resistance: where do we stand?. J Med Microb.

[12] Strahilevitz J, Jacoby GA, Hooper DC, Robicsek A (2009). Plasmid-mediated quinolone resistance: a multifaceted threat. Clin Microbiol Rev.

[13] Ghana Statistical Services (2021). Ghana Statistical Service. Ghana.

[14] Western Region Community Water and Sanitation Agency (2020). Regional profile: population, location and size. Community Water and Sanitation Agency.

[15] Vandepitte J, Verhaegen J, Engbaek K, Rohner P, Piot P, Heuck CC (2003). Basic laboratory procedures in clinical bacteriology.

[16] CLSI (2020). Performance Standards for Antimicrobial Susceptibility Testing: A CLSI supplement M100. Clinical and Laboratory Standard Institute.

[17] Chen X, Zhang W, Pan W, Yin J, Pan Z, Gao S (2012). Prevalence of *qnr, aac(6´)-Ib-cr, qepA* and *oqxAB* in *Escherichia coli* isolates from humans, animals, and the environment. Antimicrob Agents Chemother.

[18] World Health Organization (2021). Global antimicrobial resistance and use surveillance system (GLASS) report: 2021.

[19] The Lancet (2020). The antimicrobial crisis: enough advocacy, more action. The Lancet.

[20] Moges F, Eshetie S, Abebe W, Mekonnen F, Dagnew M, Endale A (2019). High prevalence of extended-spectrum beta-lactamase-producing Gram-negative pathogens from patients attending Felege Hiwot Comprehensive Specialized Hospital, Bahir Dar, Amhara region. PLoS ONE.

[21] Ghimire A, Acharya B, Tuladhar R (2018). Extended spectrum ß-lactamase (ESBL) producing multidrug resistant Gram-negative bacteria from various clinical specimens of patients visiting a tertiary care hospital. TU J Microbiol.

[22] Uc-Cachón AH, Gracida-Osorno C, Luna-Chi IG, Jiménez-Guillermo JG, Molina-Salinas GM (2019). High prevalence of antimicrobial resistance among Gram-negative isolated bacilli in intensive care units at a tertiary-care hospital in Yucatán Mexico. Medicina.

[23] Agyepong N, Govinden U, Owusu-Ofori A, Essack SY (2018). Multidrug-resistant gram-negative bacterial infections in a teaching hospital in Ghana. Antimicrob Resist Infect Control.

[24] Feglo PK, Adu-Sarkodie Y (2016). Antimicrobial resistance patterns of extended spectrum ß-lactamase producing Klebsiellae and E. coli isolates from a tertiary hospital in Ghana. ESJ.

[25] Meletis G (2016). Carbapenem resistance: overview of the problem and future perspectives. Therapeutic Advances in Infection.

[26] Iskandar K, Molinier L, Hallit S, Sartelli M, Catena F, Coccolini F (2020). Drivers of antibiotic resistance transmission in low-and middle-income countries from a “One Health” perspective: a review. Antibiotics.

[27] Labi A-K, Obeng-Nkrumah N, Dayie NTKD, Egyir B, Sampane-Donkor E, Newman MJ (2021). Antimicrobial use in hospitalized patients: a multicentre point prevalence survey across seven hospitals in Ghana. JAC-Antimicrobial Resistance.

[28] Bediako-Bowan AAA, Owusu E, Labi A-K, Obeng-Nkrumah N, Sunkwa-Mills G, Bjerrum S (2019). Antibiotic use in surgical units of selected hospitals in Ghana: a multi-centre point prevalence survey. BMC Public Health.

[29] Karikari AB, Saba CKS, Yamik DY (2020). Assessment of asymptomatic bacteriuria and sterile pyuria among antenatal attendants in hospitals in northern Ghana. BMC Pregnancy Childbirth.

[30] Odoki M, Aliero AA, Tibyangye J, Maniga JN, Eilu E, Ntulume I (2020). Fluoroquinolone resistant bacterial isolates from the urinary tract among patients attending hospitals in Bushenyi District, Uganda. PAMJ.

[31] Bediako-Bowan AAA, Kurtzhals JAL, Mølbak K, Labi A-K, Owusu E, Newman MJ (2020). High rates of multi-drug resistant gram-negative organisms associated with surgical site infections in a teaching hospital in Ghana. BMC Infect Dis.

[32] Stapleton AE, Wagenlehner FME, Mulgirigama A, Twynholm M (2020). Escherichia coli resistance to fluoroquinolones in community-acquired uncomplicated urinary tract infection in women: a systematic review. Antimicrob Agents Chemother.

[33] Bidell MR, Palchak M, Mohr J, Lodise TP (2016). Fluoroquinolone and third-generation-cephalosporin resistance among hospitalized patients with urinary tract infections due to Escherichia coli: do rates vary by hospital characteristics and geographic region?. Antimicrob Agents Chemother.

[34] Quansah E, Amoah Barnie P, Omane Acheampong D, Obiri-Yeboah D, Odarkor Mills R, Asmah E (2019). Geographical distribution of ß-lactam resistance among Klebsiella spp from selected health facilities in Ghana. TropicalMed.

[35] Newman MJ, Frimpong E, Donkor E, Opintan JA, Asamoah-Adu A (2011). Resistance to antimicrobial drugs in Ghana. IDR.

[36] Vikesland P, Garner E, Gupta S, Kang S, Maile-Moskowitz A, Zhu N (2019). Differential drivers of antimicrobial resistance across the World. Acc Chem Res.

[37] Shariff VAAR, Shenoy MS, Yadav T, Radhakrishna M (2013). The antibiotic susceptibility patterns of uropathogenic *Escherichia coli* with special reference to the fluoroquinolones. JCDR.

[38] Cardoso T, Ribeiro O, Aragão IC, Costa-Pereira A, Sarmento AE (2012). Additional risk factors for infection by multidrug-resistant pathogens in healthcare-associated infection: a large cohort study. BMC Infect Dis.

[39] Mensah-Attipoe I, Opintan JA, Newman MJ, Ashong PP (2020). Molecular characterization of ciprofloxacin resistant *Escherichia coli*from Ghana. JAMB.

[40] Ogbolu D, Alli A, Anorue M, Daini O, Oluwadun A (2016). Distribution of plasmid-mediated quinolone resistance in Gram-negative bacteria from a tertiary hospital in Nigeria. Indian J Pathol Microbiol.

[41] Goudarzi M, Azad M, Seyedjavadi SS (2015). Prevalence of plasmid-mediated quinolone resistance determinants and OqxAB efflux pumps among extended-spectrum β-lactamase producing Klebsiella pneumoniae isolated from patients with nosocomial urinary tract infection in tehran, Iran. Scientifica (Cairo).

